# A Clinical Model for the Differentiation of Suicidality: Protocol for a Usability Study of the Proposed Model

**DOI:** 10.2196/45438

**Published:** 2023-08-11

**Authors:** Remco F P de Winter, Connie M Meijer, John H Enterman, Nienke Kool-Goudzwaard, Manuela Gemen, Anne T van den Bos, Danielle Steentjes, Gabrielle E van Son, Mirjam C Hazewinkel, Derek P de Beurs, Marieke H de Groot

**Affiliations:** 1 Mental Health Institute Rivierduinen Leiden Netherlands; 2 VU University Section of Clinical Psychology, Amsterdam Public Health Research Institute Amsterdam Netherlands; 3 Maastricht University MHeNs School for Mental Health and Neuroscience Maastricht Netherlands; 4 Sussex Partnership National Health Service Foundation Trust Eastbourne United Kingdom; 5 Parnassia Mental Health Institute The Hague Netherlands; 6 Trimbos Institute Utrecht Netherlands; 7 Lentis Mental Health Institute Groningen Netherlands

**Keywords:** differentiation, suicidality, suicidal behavior, subtype, subcategory, category, categories, categorize, subcategories, validation study, mental health, suicide, suicidal, differentiation, classification, psychiatry, classify, psychiatric, suicide prevention, suicidal ideation, mental illness, suicidal thought, dying, perceptual disintegration, PD, primary depressive cognition, PDC, psychosocial turmoil, inadequate communication, intraclass correlation coefficients, ICC

## Abstract

**Background:**

Even though various types of suicidality are observed in clinical practice, suicidality is still considered a uniform concept. To distinguish different types of suicidality and consequently improve detection and management of suicidality, we developed a clinical differentiation model for suicidality. We believe that the model allows for a more targeted assessment of suicidal conditions and improves the use of evidence-based treatment strategies. The differentiation model is based on the experience with suicidality that we have encountered in clinical practice. This model distinguishes 4 subtypes of entrapment leading to suicidality. The earliest description of this model and a proposal for usability research has been previously presented in a book chapter.

**Objective:**

In this study, we present the most recent version of the 4-type differentiation model of suicidality and a protocol for a study into the usability of the proposed model.

**Methods:**

The 4-type differentiation model of suicidality distinguishes the following subtypes: (1) perceptual disintegration, (2) primary depressive cognition, (3) psychosocial turmoil, and (4) inadequate coping or communication. We plan to test the usability of the 4 subtypes in a pilot study of 25 cases, and subsequently, we will include 75 cases in a follow-up study. We looked at the case notes of 100 anonymized patients with suicidality who presented to mental health care emergency service in The Hague International Center. The summary and conclusions of the letters sent to the patients’ general practitioners after suicide risk assessment will be independently rated by 3 psychiatrists and 3 nurse-scientists for absolute and dimensional scores. The Suicidality Differentiation version 2 (SUICIDI-II) instrument, developed for this study, is used for rating all the cases. Intraclass correlation coefficients for absolute and dimensional scores will be calculated to examine type agreement between raters to examine the usability of the model and the feasibility of the SUICIDI-II instrument.

**Results:**

We consider the model tentatively valid if the intraclass correlation coefficients are ≥0.70. Subsequently, if the model turns out to be valid, we plan to rate 75 other cases in a follow-up study, according to a similar or adjusted procedure. Study results are expected to be published by the end of 2023.

**Conclusions:**

The theoretical roots of the differentiation model stem from classic and contemporary theoretical models of suicidality and from our clinical practice experiences with suicidal behaviors. We believe that this model can be used to adjust the diagnosis, management, treatment, and research of suicidality, in addition to distinguishing different dynamics between practitioners and patients with suicidality and their families.

**International Registered Report Identifier (IRRID):**

DERR1-10.2196/45438

## Introduction

Thoughts of death or suicide, planning or preparing suicide, attempting suicide, and completing suicide are defined as suicidality [1]. Although suicidal thoughts, plans, and attempts are common, completed suicide is rare. Suicidality is a symptom often found in patients experiencing mental disorder [2]. Except in case of a diagnosis of major depressive disorder or borderline personality disorder [3], suicidality is not a symptom required to meet the Diagnostic and Statistical Manual of Mental Disorders, Fifth Edition, criteria for any other psychiatric diagnosis.

Suicidality is still defined as a uniform concept [3-5], even though it is complex and multilayered and there are multiple risk and protective factors, including mental disorder, personality traits, biological factors, and psychosocial factors (to name a few) playing a role in the onset and duration of suicidality [6,7]. Scientific knowledge, such as theoretical concepts derived from neuroimaging and research into genetic vulnerability for suicide, will be difficult to apply in clinical practice [8,9], as science has not been able to distinguish different kinds of suicidality or pinpoint drivers or etiology.

It is widely accepted and acknowledged that clinical differentiation of somatic disorders has resulted in improved diagnosis and treatment strategies, for example, for breast cancer [10], diabetes [11], and dementia [12]; yet, no lucid clinical differentiation model for suicidality is available for mental health care [13], and as a consequence, general texts, suicide prevention, treatment guidelines, and scientific research hardly differentiate between various types of suicidal behaviors [14-16].

Mental health professionals confronted with suicidality are not just expected to adequately assess the suicide risk but also to manage the risk and all complexities around those risks [3]. Even when risk and protective factors are identified, the assessment of suicide risks remains complex and completed suicide is unpredictable. Professionals’ responsibility and liability are difficult to put into operation when it comes to suicide prevention, and another layer to the complexity of suicide risk assessment is added [17].

The pathway of referral to services partially defines the responsibilities of professionals involved at any point of the pathway. They do not just need to share the responsibilities for prevention of suicide with other professionals and referrers but also share this responsibility in a rational and reasonable manner with the patient or the relatives [18-20]. Everyone involved needs to be aware that not all patients with suicidality can be safeguarded by admission. Admission may protect (temporarily) against suicide but can engender an iatrogenic effect, resulting in maintaining the suicidal state rather than reducing it, which is unwanted [21,22]. This is why the emphasis of the management of patients with suicidality is often focused on safety planning and, if applicable, the treatment of underlying mental health disorders.

The complex dynamics around the risks resulting from suicidality and the focus on safety may lead to formalized and restrictive defensive practice. This is why we believe that it will be helpful to discern different types of suicidality and consider to what extent the patient with suicidality is able to take responsibility for their own safety during recovery from a suicidal condition. Differentiation may also help to determine which treatment conditions could be more tailor-made to diagnosis and treatment in mental health. The differentiation of suicidality may encourage practitioners from various networks to apply clear unambiguous language about suicidality.

The result of the scientific quest for suicidal typologies for clinical practice in mental health care was the development of several theoretical models [23-30]. Those models offered improved insight into the complex processes leading to suicide [29-31]. Classical, contemporary, and empirical typologies of suicide have been established [13] and were important for the development of the model under investigation. For instance, the Integrated Motivational-Volitional Model of Suicidality [31] distinguishes between the onset of suicidal thoughts and the dynamic process of engaging into suicidal acts. However, existing theoretical and empirical typologies of suicidality have limited use [3] in clinical practice for various reasons as follows:

1. The application of suicide typology, which is based on variables not related to entrapment, may result in unreliable suicide risk estimates.
2. The context in which suicidality appears—next to theoretical and empirical typologies—determines whether specific, individual factors increase or mitigate the suicide risk [32]. For example, unemployment, a risk factor for suicide, may result in sudden and quick increase in suicide risk when a breadwinner of the family is laid off, whereas someone with long-term unemployment may experience chronic suicidality due to longstanding exposure to stress, substance abuse, and depression. For people who find it hard to maintain themselves in a work environment, unemployment can be a blessing in disguise and as such may become a protective factor for suicidality.

**Figure 1:**
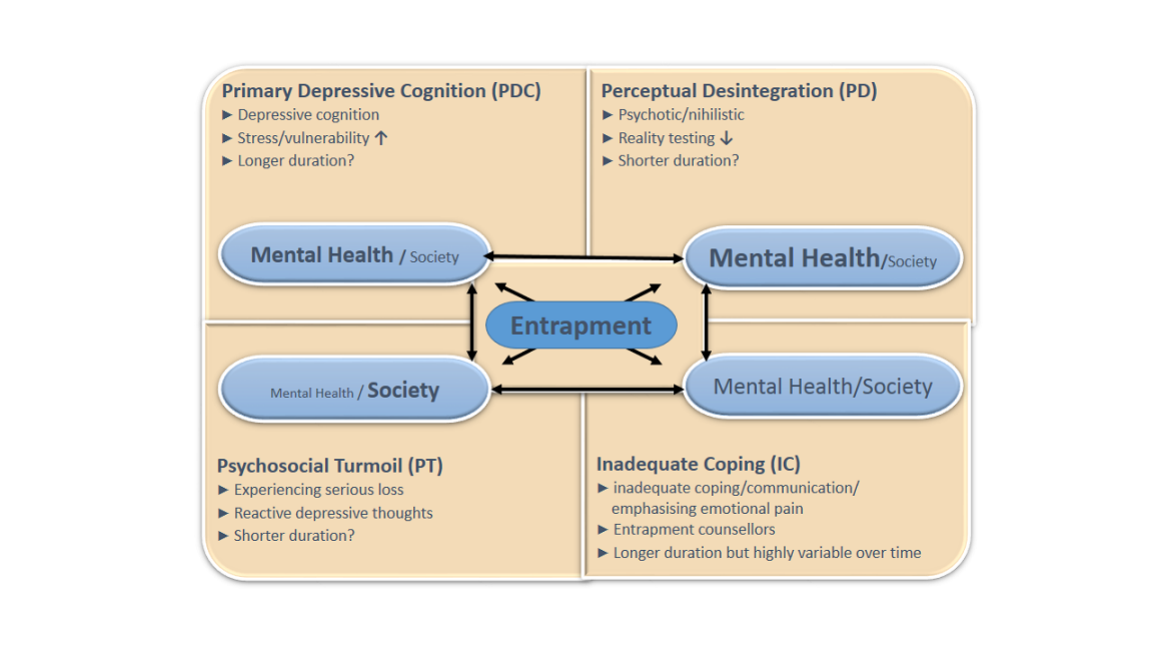
The 4 subtypes of entrapment of suicidality and theoretical aspects.

The experience of entrapment seems to play a crucial role in the etiology of suicidality as also described in the integrated model of stress vulnerability [33,34] and entrapment [35], which has been developed for the Dutch suicide prevention guideline [16]. The principles of the entrapment theory [23] of suicidality were integrated in the current differentiation model. Entrapment is a mental state in which a person is trapped in the perception that escape is possible by ending their life. We hypothesize that there are 4 ways in which someone might become entrapped. We hypothesize there may be overlap between those paths. We developed a clinical differentiation model for suicidality: the (hypothetic) 4-type model of entrapment of suicidality (h4ME; Figure 1). This model describes 4 subtypes of entrapment of patients with suicidality, with or without mental disorder. Many scientists and practitioners were involved in the development of the h4ME model [3]. The model was revised in 2 Delphi rounds with psychiatrists, psychologists, and mental health nurses who were not involved in the proposed study. The first meeting was in March 2017 with a selection of psychiatrists, people with lived experience with suicidality, peer supporters, nurses, and psychologists employed by Parnassia Mental Health Institute. Feedback from participants of the meetings was provided by email. The second meeting was held during the Dutch Annual Conference of Psychiatrists in 2018 and was attended by psychiatrists only [36]. Feedback from attendees was provided by email.

Another contributor to the development and evolution of the h4ME model was the Dutch Multi-Disciplinary Guideline for the Assessment and Treatment of Suicidality [16] and the Professionals In Training to STOP suicide study [37] examining the effect of an e-learning “train the trainer model on education” and training of mental health care professionals in the application of the guideline principles [38]. Mental health care professionals are trained to assess suicidality in accordance with the Clinical Assessment of Suicidal Episodes [39] to assess to what extent the patient feels trapped (entrapment); the stronger the feeling of being trapped, the higher is the suicide risk [35]. This inspired us to differentiate between etiologies of entrapment (Textbox 1). We hypothesize that all kinds of suicidality encountered in clinical practice (as well as cases of completed suicide) can be assigned to one of the following 4 types [40]:

1. Perceptual disintegration (PD): entrapment originating in the context of disturbed perceptions or (affective) psychotic behaviors
2. Primary depressive cognition (PDC): entrapment in the context of (a) depressive cognition(s)
3. Psychosocial turmoil (PT): entrapment in the context of acute reactivity to a (perceived or actual) loss, offence, adversity, or doom
4. Inadequate coping or communication (IC): entrapment in the context of communicating intense distress or emphasizing emotional pain

It is not known whether this model will encompass the full range of suicidal behaviors occurring in mental health care services though. To optimize the assessment of suicide risks and the care of patients with suicidality, it is necessary to test the usability of this model before it is applied in clinical and research practice (Textbox 2). The objectives of our intended study are as follows:

1. Investigate the usability of the h4ME model—examine whether the h4ME model accurately describes the complete spectrum of suicidality as encountered in specialist mental health care services.
2. Examine whether the Suicidality Differentiation version 2 (SUICIDI-II) instrument allows clinicians to assign cases of suicidality to subtypes as described in the h4ME model.
3. Investigate whether the h4ME model and the SUICIDI-II should be adjusted if it appears that subtypes of entrapment overlap.

**Descriptions of the 4 subtypes of suicidality (use of narcotics or other substances or somatic symptoms can be viewed as adjustors of which the effect depends on the differentiation of suicidality).**

**Primary depressive cognition**

Suicidality stems primarily from a depressive thought process and there are no psychotic features (yet). The depressive state can be present for a while (eg, weeks or months). Thoughts of suicide, which are part of the cognition and present on a daily basis, are characteristic. There is clear evidence of distress, which can be noticed by the examiner because of the depressive thought process. A classic example would be a depressive disorder, but primary depressive cognition may also be part of an anxiety disorder, autism, etc. The features of a personality disorder may be mixed with the depressive state, or the depressive state may be caused by a personality disorder and become part of a returning thought pattern in which negative cognitions and Beck cognitive triad can be present (negative views about oneself, negative views about the world, and negative views about the future).

**Perceptual disintegration (psychotic disturbed perception or behavior)**

Suicidality originates from psychosis, which can often be accompanied by affective (depressive) dysregulation or can be affected by it. Usually, the psychotic state has only been present for probably a short time (rather days or weeks than months) and is noticed (or becomes apparent) because of its severity. Suicidality may originate from depressogenic cognition; however, in that case, the severity has developed to such a level that it can be seen as a mood-congruent or mood-incongruent psychotic state. The distress can be understood, but the severity cannot be perceived as comprehensible anymore by the examiner. A classic state is a depression with mood-congruent psychotic features. However, it can also appear among people who, while in a psychotic state, are ordered by their delusions to hurt themselves.

**Psychosocial turmoil**

Suicidality stems primarily from a severe loss or blow to the ego, leading to a complete upheaval of someone’s life. The person experiences enormous guilt, severe shame, or does not dare to look another in the eye anymore or experiences a downfall without being in a psychotic state. There is an unbearable anguish, which leads to a need for release from that pain or the need not to exist anymore, to not be able to feel or escape the awful misery or pending dread. Usually, someone has been in this state for a short time (hours, days, or weeks). Drug use can be extra provoking. The stress is perceivable for the examiner from the perspective of loss or a blow to the ego and there may be slight psychotic features, but one can follow the narrative. Underlying dysregulation of the impulsivity can worsen the state and increase the risk of a lethal outcome.

**Inadequate coping or communication**

Suicidality stems from a severe feeling of distress and not being able to communicate this properly. There is difficulty with formulating an adequate request for help and one seems to be hoping for a solution by demonstrating suicidality. This behavior usually exists for a longer period (months) and fluctuates severely. This type of a more chronic suicidality is often seen as part of a personality disorder such as a borderline personality disorder. Also, drug use can be an important provoking factor. Suicidality is perceived by others as “externalizing” and fake and can result in aid workers feeling “trapped” in the dynamics. The behavior can coincide with experiences of loss with which the powerlessness is externalized and not internalized. Often, the support system is exhausted and professionals are viewed as failing. The major risk is for professionals to feel manipulated and for the person who is assessed to feel misunderstood and not taken seriously, which leads to an amplification of the behavior, accompanied by an increased risk of suicide. Contrary to how it is perceived by others, the person is genuinely distress. Suicide can be used as the ultimate way to communicate about the distress caused by the perceived unfair or rejection judgment of the person (especially recognizing and exploring the countertransference and offering help to the underlying motivators of suicidality are essential with this type).

**Examples of conclusions and vignettes of entrapment of suicidality typology.**

**Vignette 1: Perceptual disintegration**

This case concerns a mental health act assessment of a 25–29-year-old woman with no history of mental illness, except for a previous one-off assessment. She has 2 (biological) children younger than 4 years of age. She came to the notice of the police when she—in company of the children—started to ring the bells at the houses of total strangers after a 1-sided car accident (total loss) and expressed suicidal and homicidal thoughts, leaving the children behind in a confused state. People involved in the incident were shocked by the bizarre presentation. At the time of the mental health act assessment, we saw a very tense woman who was clearly trying to keep up a facade and could not reason or answer questions adequately. The presentation was suspect of a paranoid state, which possibly already existed for some time. During the police investigation, her statements were bizarre, for example, mentioning that she was murdered. There was some suspicion of substance abuse. The assessment could not confirm direct symptoms of acute suicidality; however, taking into consideration the events, earlier statements, and the ensuing silence, suicide risk was assessed as acutely increased. Because she showed no insight and refused voluntary admission, an involuntary admission was arranged and agreed. The children were placed with foster parents by social services and child and family services.

**Vignette 2: Primary depressive cognition**

This case concerns a 50–54-year-old man who was referred by the mental health nurse working in the general practitioner practice. He was referred for an emergency assessment within the community team because of low mood and suicidal ideation. He had consistent ideas of different ways to kill himself, though he considered himself a coward. During the assessment, we saw a depressed man with low self-esteem who normally pushes away emotions. After a small incident at work, he completely broke down and has been on sick leave for the last 4 weeks with low mood, anhedonia, ruminations, and sleep problems. Fifteen years ago, he experienced a similar episode and at that time, he did a suicide attempt with medication and alcohol after the death of his father. At the time, he was referred and treated by the community team. Six years ago, he had a myocardial infarction. He was diagnosed with depression. It was possible to agree to a safety plan, and the suicide risk was considered not to be acutely increased. The patient was referred to the community team—with a safety plan in place—for the treatment of depression.

**Vignette 3: Psychosocial turmoil**

This case concerns a home assessment of the suicide risk of a 20–24-year-old woman with no previous psychiatric history. The general practitioner asked for an assessment after she made suicidal statements following several serious and negative life events over the last few weeks (relationship breakup, termination of pregnancy, debts, death of grandfather, suicide of friend, loss of accommodation). During the assessment, we saw a tense, desperate woman with insufficient coping strategies to manage the situation, becoming overwhelmed as a result. She was unable to pull herself out of the situation and feels so miserable that she does not have any hope of a good outcome. She expressed suicidal ideas and her support system was unable to support her. Her limited coping skills were possibly due to a disturbed personality development and below average intelligence. A respite admission (time out admission) was indicated to stabilize this patient and work toward follow-up treatment in the community. It was not possible to arrange admission or involve intensive home treatment because of limited capacity within those services, though it was possible to set up a safety plan until the next morning and arrange for alternative follow-up care in the community.

**Vignette 4: Inadequate coping or communication**

This concerned a suicide-risk assessment at “services for acutely disturbed people” of a 45–49-year-old female who wanted to be addressed as male, without a gender reassignment or transformation having taken place yet. They are known with posttraumatic stress disorder, personality problems, autism spectrum disorder, gender dysphoria, and dissociative episodes. In the past, they attempted suicide on several occasions and autoamputated fingers and toes. Patient was discharged from the admission wards 7 days ago or 7 days before the current assessment. Specialized in-patient treatment for this patient with severely disturbed behavior was terminated because adequate treatment was not possible due to splitting, dismissing, and devaluating the treatment plan, projection, and denial demonstrated by the patient. Patient now comes to the attention of community mental health services, referred by the police, after they made serious suicidal statements and threw the phone down when called by the crisis services. The crisis team contacted the police, who found the patient near a canal, in possession of a knife. During the assessment, the patient displayed complex, claiming behavior possibly because she was unable to acquire Dormicum from the assessing team. Prescription of Dormicum was denied because of the inappropriateness of the request. Patient stated they cannot agree to a safety plan a long as they are not provided with Dormicum. Admission was not considered to be suitable because of the recent discharge and the lack of cooperation with the proposed treatment plan in the patient ward. The outcome of the assessment was to send the patient home and contact the responsible professional of the community team. When the outcome was communicated with the patient, they decided to agree with some form of a safety plan.

## Methods

### Design: Explorative Qualitative Study

#### Sampling

In a previous study conducted at The Hague Emergency Service, one-third of the assessed patients exhibited suicidality [41,42]. In this study, 100 cases of patients with suicidality were included in the order of entry. For the pilot study, 25 cases will be included. Subsequently, feedback from raters is collected and procedures may be adjusted. Then, 75 other cases (numbered 26-100) will be rated. The identity of the referrer, the patient, the (nurse) practitioner, and the general practitioner cannot be deduced from the results and conclusions.

#### Raters

Raters (n=6) will be recruited from RFPdW’s professional network (3 psychiatrists and 3 registered nurses); all are employed at psychiatric emergency services. They all have extensive knowledge and experience in assessing suicidality due to clinical or scientific positions outside The Hague Emergency Service. The raters are not involved in any of the cases included in this study. RFPdW will not be a rater. Raters will be asked to independently assign cases to entrapment types by using the SUICIDI-II, an instrument especially developed for this study to investigate to what extent raters agree on the entrapment type that should be assigned to each case (further indicated: type agreement [TA]). Before scoring, raters will be trained in the h4ME model and in using the SUICIDI-II.

### The SUICIDI-II: An Instrument for Assigning Cases to Entrapment Types

To determine TA, the SUICIDI-II was designed for this study. The SUICIDI-II should be considered a preliminary systematic description of the 4 entrapment types. From 2018 onward, the SUICIDI-II and previous versions and the h4ME model were presented and discussed during conferences in the context of suicide prevention in the Netherlands and abroad [19,36]. The feedback of the attendees was collected during the meetings. The feedback was processed by RFPdW and MHdG and led to adjustments of subsequent versions of the SUICIDI. The SUICIDI-II describes each entrapment type by 3 propositions. The propositions are hierarchically formulated to indicate whether a type is applicable or not. Scores are rated as 0=type is not applicable, 1=type leaves room for other entrapment types, and 2=matches type and excludes other types. See the following link [43] for the SUICIDI-II questionnaire.

### Outcome Measures

TA will be investigated in two ways: (1) by calculating an absolute TA (aTA) score and (2) by two different dimensional agreement scores (d1TA and d2TA). The dimensional scores were introduced because we expect that cases might be assigned to more than 1 subtype. aTA will be examined by asking raters to allocate each case to only 1 entrapment type (type PD, PDC, PT, or IC). This will result in an aTA score. Dimensional TA is scored as follows:

1. d1TA: raters are asked to allocate a total of 4 points to one or more subtypes (minimum 0 to maximum 4 for a subtype); the higher the allocated score, the more a subtype applies.
2. d2TA: raters indicate which proposition (1 or 2) most likely applies to the types they scored for the d1TA as proposed in the SUICIDI-II instrument. Subtypes that were not chosen automatically scored 0 (=not applicable).

### Data Analysis

Intraclass correlation coefficients (ICCs) will be calculated to quantify the degree of agreement between the raters on the assigned subtypes: aTA, d1TA, and d2TA. Differences between measurements can be due to real differences (between persons or within persons on repeated measurements) or from noise (differences due to imperfections in the description of the types). This is why we will also calculate an ICC for the extent to which raters agree on which statement (1 or 2) best describes the chosen type (d2TA). In SPSS (version 23.0; IBM Corp), the analyses will be performed with a 2-way mixed-effects model, aTA, according to the guidelines of selecting and reporting ICC [44]. ICCs are numbers between 0.0 and 1.0 with a 95% CI. In a perfect model, when ICC=1, all differences are completely real. In a completely invalid model, all differences are noise and ICC=0. In other words, the higher the ICC, the more the raters agree. An ICC<0.50 is indicated as poor, 0.50-0.75 is indicated as moderate, 0.75-0.90 is good, and >0.90 is excellent [44]. The model is considered valid if the ICC for aTA and the 2D TAs (d1TA and d2TA) are ≥0.70. The model will be adjusted if the ICC or the aTA or d1TA are ≤0.70. The propositions in the SUICIDI-II are considered valid usable if the ICC of d2TA is >0.70 and will be adjusted if the ICC is ≤0.70.

### Schedule

The first step is to carry out a pilot study in which we examine the usability of the h4ME model of suicidality. We aim to answer the following questions:

1. Is a selection of mental health care workers capable of dealing with the h4ME model and the SUICIDI-II instrument?
2. Can conclusions from patient records of high-risk patients with suicidality assessed by the outreach psychiatric emergency services be used for rating absolute and dimensional TA?
3. Are the proposed subtypes (PD, PDC, PT, and IC) validly definable when various clinicians independently allocate cases to subtypes? How are subtypes distributed?
4. Are these subtypes dimensionally delineated by using 2 different modes of scoring, and is there consensus when different clinicians independently score them? What is the reliability of the different modes of scoring?
5. Which form of dimensional scoring is preferred?
6. If applicable, how can we improve the SUICIDI-II instrument?
7. What feedback can we provide to raters when there is any indication that raters scored incorrectly?

Step 2 is a follow-up study of 75 cases according to the same (or eventually adjusted) procedure. Further, overlap between subtypes will be investigated.

### Ethical Considerations

The Medical Research Ethics Committee Leiden the Hague Delft involving the Human Subjects Act (*Wet medisch-wetenschappelijk onderzoek met mensen*) was consulted prior to the start of this study. The committee decided in 2020 that no approval was needed (G21.021/PV/pv). The medical directorates and privacy officers of the Mental Health Institute Rivierduinen and Parnassia Mental Health Institute approved the study, and both institutes financed the study [3].

## Results

Between January 2018 and January 2020, a total of 503 cases of patients with suicidality were examined and assessed by the outreach psychiatric emergency service of The Hague in the Netherlands [42]. Of the 503 cases, 100 were included in this study in the order of entry. RFPdW was medically responsible for the cases. Cases were anonymized, and summarized conclusions were taken from the general practitioners’ discharge letters, which were sent after the patients’ assessment. Patients gave consent to be included in this study. The letters to the patient’s general practitioners are saved in the electronic patient record and were cosigned by RFPdW. The summarized conclusions were copied from the electronic patient record and pasted into a Microsoft Word file. These were distributed among raters. We have elaborated previously on risk management and treatment algorithms (including pharmacological and psychological interventions) across the 4 types (see Table 1), as described before in a book chapter of de Winter et al [3], and revised it.

The results of our study are expected to be published by the end of 2023. Findings of this study will be published in peer-reviewed journals and presented at regional, national, and international professional conferences and meetings.

**Table 1:** Hypothetical risk management and treatment algorithm

	Perceptual disintegration	Primary depressive cognition	Psychosocial turmoil	Inadequate coping or communication
Severity of the suicide risk	++++^a^	++	+++	+
Duration	Days or weeks	Weeks or months	Days	Days or hours; often exacerbation of chronic suicidality
Influence culture	+	++	++++	+++
Religion	+	++	++++	+++
Spiritual affiliation	+	++	++++	+++
Economic conjuncture	+	++	++++	+++
Social disorder (war, pandemic, etc)	+	++	++++	+++
Live events or loss experiences	++	++	++++	+++
Genetic biochemical factors	Yes	Yes	Maybe	Probably
Influence personality	+	++	+++	++++
Major life events	Yes	Yes	Yes	Yes
True mental disorder	Yes	Yes	Maybe	Mostly
Expected course	Reduction after treatment of psychosis	Reduction after biological and psychological treatment	Reduction when tunnel vision decreases, reduces when peak of mourning has passed	Nonspecific reduction within hours or days or when behavior has been exposed or when underlying problems have come to the surface, risk of acute shift from chronic risk and shift to another type
Recurrence	New psychotic episode, triggering of trauma	Recurrent affective disorder	Recurrent episode of psychosocial stress or continuation of severe stress, received narcissistic affront	Interpersonal stress and perceived powerlessness, lack of external recognition of underlying distress
Reassessment of suicide risk	Several times a day, continuous during treatment, after recovery, with the recurrence of a new episode as precaution during trauma therapy	Several times a day, regularly during treatment, after recovery, new episode when the mood deteriorates	Several times a day, ranging from a few times a day to often, in the aftermath of an acute suicidal episode, during a new episode of severe psychosocial stress or new setback	After the suicidal episode, when continued or renewed lack of recognition of underlying distress, during interpersonal stress and perceived powerlessness
Pharmacotherapy	Antipsychotics (clozapine) or mood stabilizer (lithium), possibly additional benzodiazepines in the event of major anxiety	Antidepressant or mood stabilizer (lithium), restrained use of benzodiazepines when increased risk of impulsivity, short-term benzodiazepines for sleep deprivation	Restrained use of medication, possibly symptom relief for sleep deprivation or great anxiety	Hold back medication when possible (changes in or addition to) pharmacological treatment
Actions during crisis	Admission (if needed), intensive home treatment if risk is acceptable	Emergency care, intensive home treatment	Organize mourning, support from family and relatives, brief admission	(F)ACT, crisis plan, maintain autonomy
Relatives	Involving relatives for discussing acute risk, safety, and treatment	Involving relatives for safety and treatment	Involving relatives for direct support and interaction	Involving relatives more for exploring dynamic interactions
Follow-up	Outpatient treatment of psychotic symptoms, trauma treatment	Outpatient treatment of depressive symptoms with cognitive behavioral therapy, Collaborative Assessment and Management of Suicidality, Eye movement desensitization and reprocessing	General practitioner	(F)ACT^b^, additionally, DBT^c^ or Collaborative Assessment and Management of Suicidality or collaborative care; eye movement desensitization and reprocessing; vigilant for change of symptoms
Responsibility of the patient	Increasing when disintegration reduces	Increasing when depressive symptoms reduce	Increasing when “tunnel vision” fades	Retain as much responsibility as possible but be careful by disstress
Responsibility of the caregiver	Decreasing when disintegration reduces	Decreasing when depressive symptoms reduce	Decreasing when “tunnel vision” fades	Holding back of taking over control; offer maximum support; recognize emotional distress

^a^More + symbols indicate greater influence or risk.

^b^ACT: Assertive Community Treatment.

^c^DBT: Dialectical behaviour therapy.

## Discussion

### Principal Results

The h4ME model, whose usability will be examined in this study, has been designed on the basis of clinical experience with suicidal behavior. Rather than taking the usual route of applying theoretical knowledge to practice, we took the reverse route by proposing a theoretical model based on practical experience [3]. We differentiated appearances of suicidal behavior into types of entrapment—the mental state in the way someone feels trapped in their belief that dying is the only way to escape from distress. In this model, we identified and described 4 different pathways to entrapment. We hypothesize that it depends on the entrapment mode whether the patient is able to take responsibility. We believe that the management of suicidality in accordance with the h4ME model may improve suicide risk assessment; enhance the application of evidence-based treatment strategies; and will be supportive in research of suicide prevention strategies whether it is biological, psychotherapeutic, or social research [2,45-49] or research of effective implementation of suicide prevention tools in clinical practice.

For professionals, the practical management of acute suicide risks is a priority rather than appraising theoretical or scientific concepts about the etiology of suicidal behavior. They may benefit from a theory-based but accessible framework similar to the h4ME model, which is eclectic and based on a fusion of well-known and generally accepted theoretical concepts of suicidality. The main inspiration for the model includes concepts of “dimensions of psychopathology” and the “temperament and character inventory.” This model incorporates 2 subtypes of the psychopathology dimension: PD and affective dysregulation (PDC) [50]. The remaining subtypes PT and IC are derived from the “Temperament and Character Inventory” [51], stating that there may be a relationship between personality dimensions, including 2 out of 3 dimensions of character (self-directedness and cooperativeness) and 3 out of 4 dimensions of temperament (harm-avoidance, novelty seeking, reward dependence). Differentiation may provide better insight into etiological relationships between underlying psychological or biological dysregulation processes. We estimate that in the future, the model can be used to determine which available evidence-based treatment strategies are most promising for the various subtypes. For instance, we suggest that the patients with suicidality of subtypes PD and PDC may recover with the treatment of the underlying mental disorders, whereas patients with subtype PT or IC may take advantage of treatment focusing on strengthening coping skills.

We assume that practitioners using the model will find it less complicated to consider suicidality and to put the risks of suicidality in context, and patients with suicidality may feel more comfortable when practitioners better understand patients’ motives to contemplate suicide. Additionally, the model can be used to determine the best fitting of a suitable treatment strategy. Finally, application of the model for scientific research may lead to the discovery of novel and more effective treatment strategies and ultimately to more effective suicide prevention. We do not rule out that the 4 proposed types, especially in the context of effective treatment, might be more defined and specified. Additionally, we do not rule out that more subtypes can be identified in clinical and nonclinical populations.

### Limitations

The development of the h4ME model is a venture into unknown territory. Little is known of the nature and origin of suicidality, so the model does not provide explanations or answers to the many questions about the onset of suicidality. A limitation of this study is that cases who will be included were willing to seek help. We are convinced that this population does not represent all kinds of people with suicidality, especially those who refuse help or hide suicidal ideation. This may affect the outcomes. In addition, we identified 4 subtypes, automatically excluding possible other types. For instance, people experiencing declining somatic health, loneliness, and existential questions around the end of life are not represented in the model and consequently cannot be rated. This may also affect the outcomes, as raters are forced to indicate a limited spectrum of suicidality, and this may increase the chance that a high level of TA will be achieved. The anonymized cases and summarized conclusions taken from general practitioners’ discharge letters used for this study may be biased because they contain subjective information, written by a clinically experienced nurse and a junior doctor. There may be distortion of the real suicidal symptoms due to a variety of factors such as countertransference, a diversity of professional backgrounds, or the lack of clinical experience.

### Suggestions for Further Research

The majority of suicide cases occur outside the reach of mental health care services. Research among these groups is likely difficult because of privacy and ethical issues. Psychological autopsy studies of cases of suicide who are not accessed by mental health care may be useful for further development or fine-tuning of the model. It may reveal a different distribution of types, additional types, and specific information about entrapment pathways, for instance, whether certain types are preceded by other types in the model or whether certain types are the superlative of another type. In future studies also, Likert-scale items reflecting psychopathological symptoms, behaviors, thoughts, emotions, duration, temperament, and character may be used for the eventual differentiation of subtypes. It is important to include other mental health care workers—other than psychiatrists and nurses—in future validity research. It would also be useful to include multidisciplinary teams (including psychiatrists, nurses, nurse practitioners, psychologists, general practitioners, and social workers) as clusters of raters; these teams all may contribute to adjustment and usability of the model and the feasibility of the SUICIDI-II instrument. The usability strategy may be adjusted in future studies. Finally, correlations between subtypes and demographic, context, and (mental) health variables might be investigated.

### Conclusions

One of the most poignant results of this model is that the differentiation of professional responsibilities has emerged. Improved clarity about those professional and personal responsibilities allows a more evidence-based discussion, for instance, about working as team, about sharing responsibilities, or supporting others when needed. Ultimately, this is what makes the model truly stand out and gives it added value. The model and the research proposal are in its infancy. So far, the model has been considered useful by clinicians who have rated it or worked with it. It is found to be practical and easy to apply. We welcome feedback, critical considerations, discussions, and suggestions from clinicians, scientists, researchers, and policy makers.

## Abbreviations

aTA: absolute type agreement
h4ME: (hypothetic) 4-type model of entrapment of suicidality
IC: inadequate coping or communication
ICC: intraclass correlation coefficient
PD: perceptual disintegration
PDC: primary depressive cognition
PT: psychosocial turmoil
SUICIDI-II: Suicidality Differentiation version 2
TA: type agreement

## Data Availability

The data sets generated during this study are publicly available as an SPSS file [52]; all parameters (for some that are traceable to an individual) have been coded. The keys for the coded parameters are available from the corresponding author on reasonable request.
